# Evaluating real-world performance of an automated offline glaucoma AI on a smartphone fundus camera across glaucoma severity stages

**DOI:** 10.1371/journal.pone.0324883

**Published:** 2025-06-26

**Authors:** Sirisha Senthil, Divya Parthasarathy Rao, Florian M. Savoy, Kalpa Negiloni, Shreya Bhandary, Raghava Chary, Garudadri Chandrashekar

**Affiliations:** 1 VST Center for Glaucoma Care, LV Prasad Eye Institute, Hyderabad, India; 2 Remidio Innovative Solutions, Inc, Glen Allen, United States of America; 3 Medios Technologies, Remidio Innovative Solutions, Singapore; 4 Remidio Innovative Solutions Pvt Ltd, Bengaluru, India; Alexandria University Faculty of Medicine, EGYPT

## Abstract

**Purpose:**

Leveraging an artificial intelligence system (AI) for glaucoma screening can mitigate the current challenges and provide prompt detection and management crucial in averting irreversible blindness. The study reports the real-world performance of a glaucoma AI system deployed on a smartphone-based fundus camera across various severities of glaucoma.

**Methods:**

In this prospective comparative study at a tertiary care glaucoma clinic, consecutive patients were evaluated by a glaucoma specialist using clinical assessment, visual field tests, and SD-OCT, and categorized as definite glaucoma, glaucoma suspect, or no glaucoma. For glaucoma patients, severity was determined using Hoddap-Parrish-Anderson criteria based on visual field mean deviation (MD). A disc-centered image per eye was captured using a validated portable non-mydriatic fundus camera. The AI tool’s ability to detect referral-warranted glaucoma (glaucoma and glaucoma suspects) versus no glaucoma was compared to the specialist’s diagnosis.

**Results:**

We included 213 participants with a mean age of 55 ± 14.7 years (18, 88). The glaucoma specialist diagnosed 129 subjects as definite glaucoma (early-23, moderate-31, severe-75), 33-disc suspects and 51 as no-glaucoma. The automated AI system based on fundus images achieved an overall diagnostic accuracy of 92.02%, sensitivity of 91.36% (95%CI 85.93% to 95.19%) and specificity of 94.12% (83.76% to 98.77%) for referral warranted glaucoma. The 14 false negatives included 5-disc suspects and 9 definite glaucoma (3-early, 3-moderate and 3-advanced glaucoma). The sensitivity of AI for detecting early, moderate and advanced glaucoma was 86.9% (95%CI 66.4–97.2), 90.3% (95%CI 74.3–97.96), and 96% (88.75% to 99.17%) respectively.

**Conclusion:**

In a real-world setting, the AI-based offline tool integrated on a smartphone fundus camera showed a promising performance in detecting referral-warranted glaucoma compared to a glaucoma specialist’s diagnosis. The AI showed higher accuracy in detecting advanced glaucoma followed by moderate and early glaucoma.

## Introduction

Glaucoma, a progressive optic neuropathy, is the leading cause of global irreversible blindness and is typically asymptomatic till its advanced stages [1]. The worldwide prevalence of glaucoma among individuals aged 40–80 years was estimated to be 3.54% (with a 95% confidence interval of 2.09% to 5.82%), equating to approximately 1 in 30,000 people at risk of glaucoma [2]. The projected numbers are expected to continue to rise alarmingly, more so in Asian countries [3]. A meta-analysis on the global extent of undetected glaucoma in adults revealed that, on average, over half of the glaucoma cases globally are undetected and this increases to over 90% in developing nations [4]. This underscores the urgent need for strategies for early detection and prevent a surge in visual disability and blindness resulting from glaucoma.

Glaucoma is characterised by functional impairment and structural alterations, primarily affecting the optic nerve head (ONH) and the retinal nerve fibre layer (RNFL) [1]. Most of the patients are generally asymptomatic at the early stages of glaucoma till the central vision gets affected in the advanced stages. It has been observed that as the severity of glaucoma increases, vision-related quality of life tends to deteriorate, with advanced stages exhibiting poorer outcomes when compared to mild and moderate glaucoma [5,6]. The detection of glaucoma is a complex, subjective, time-consuming process hinging on various examinations and needs clinical expertise. Imaging techniques play a pivotal role in assessing structural irregularities. Monoscopic fundus photography has demonstrated similar diagnostic accuracy for glaucoma detection compared to stereoscopic photography [7,8]. Hence, using monoscopic fundus image-based screening for detecting glaucomatous changes proves to be practically beneficial.

Artificial intelligence using fundus images has been well-established for screening diabetic retinopathy, AMD, cataract, ROP, refractive error and glaucoma [9,10]. While there have been several groups that have developed promising AI models for glaucoma screening based on optic disc evaluation, there is limited data on prospectively validating these models [11–20]. The performance and utility of this innovative offline AI tool on a smartphone-based fundus camera to detect referable glaucoma was earlier reported [21–23]. This investigation explores a new dimension that has not been evaluated in existing studies. The study aims to assess the diagnostic ability of this novel AI tool in a real-world setting based on optic disc images to detect referral-warranted glaucoma and we also aim to evaluate its performance in various severities of glaucoma. The diagnostic ability of the AI device is tested against the in-clinic diagnosis made by a glaucoma specialist following a thorough clinical and diagnostic evaluation. This study evaluates a portable, user-friendly device for detecting referral-warranted glaucoma.

## Materials and methods

This prospective, observational study was conducted at the glaucoma clinic of a tertiary care institute in South India between 29 September and 30 December 2022. The study was approved by the Ethics Committee (LEC-BHR-P-08-22-919) and adhered to the tenets of the Declaration of Helsinki. Written informed consent was obtained from all the participants. This study was registered under the Clinical Trials Registry of India (CTRI) – details REF/2022/10/058951.

The primary focus of this study was to evaluate the performance of an offline glaucoma AI tool integrated on a smartphone-based fundus camera against the expert’s diagnosis on different severity levels of glaucoma (early, moderate or advanced). Consecutive patients >= 18 years who consented to participate in the study were recruited. These included patients diagnosed with glaucoma (definite glaucoma and disc suspects) and controls (no glaucoma). Glaucoma group included primary open angle glaucoma (POAG), primary angle closure glaucoma (PACG), juvenile open angle glaucoma (JOAG), Pseudoexfoliation glaucoma (PXG) and steroid induced glaucoma (SIG). The details of exclusion criteria are described in S1 Appendix in S1 File All the participants underwent a comprehensive eye examination, glaucoma evaluation and imaging using the study device along with glaucoma AI output.

### Clinical examination

The participants underwent comprehensive eye examination which included refraction, best-corrected visual acuity (distance and near), intraocular pressure (Goldman applanation tonometry), gonioscopy (Sussman 4-mirror), anterior (slit lamp) and posterior segment (78D or indirect ophthalmoscopy) examination. Additional investigation for those diagnosed with or suspected of having glaucoma included, visual field examination (Humphrey HVF 24−2 or 10−2), SD-OCT (ONH and macular cube), along with fundus photography. Any unreliable visual field reports (>20% rate of fixation loss or >15% false-positive/false-negative), poor OCT signal strength (<6), significant media opacity and other ocular pathology that affected the clear imaging of the fundus were excluded (S1 Appendix in S1 File). Following dilation, the severity of cataract was graded based on the Lens Opacification Classification System (LOCS III) [24]. All the respective clinical investigations were conducted by experienced optometrists and the final diagnosis/management of the patient was by a glaucoma specialist (experience >15 years). The glaucoma specialist provided a final clinical diagnosis based on structural and functional correlation using optic disc examination, SD-OCT and visual filed examination findings as “definite glaucoma”, “disc suspect” or “no glaucoma” (S2 Appendix in S1 File). For those diagnosed with definite glaucoma, the severity of glaucoma was categorized as “early”, “moderate” or “advanced” based on Hoddap –Parrish –Anderson criteria [25].

### Fundus imaging

We used a smartphone-based non-mydriatic portable fundus camera (Remidio Fundus on phone, FOP NM 10) with an integrated offline glaucoma artificial intelligence algorithm. The imaging protocol included capturing a disc centered image (42-degree field of view) for each eye before pupillary dilation. The inbuilt automated quality check outputs the quality of the image captured as “sufficient” or “insufficient”. If the image quality was insufficient, the operator was alerted to repeat image capture (two more attempts were allowed) to obtain a sufficient-quality image. These images were analyzed by the AI. The AI outputs the vertical cup-to-disc ratio (vCDR) for each eye, along with a patient-level screening report based on structural changes in the optic nerve head (ONH) and retinal nerve fiber layer (RNFL). The final report classifies the patient as having referable glaucoma (urgent referral), being a disc suspect (suggested referral), or having no referable glaucoma (yearly follow up), based on the worse-eye and determines the urgency for referral when needed. If the image quality was insufficient (despite 3 attempts), then the eyes were dilated with 1% tropicamide solution and the images were acquired. In another group of participants, dilated fundus images were captured in those that were referred from other services or for a visual field evaluation in the glaucoma clinic. The pupil status in which the image was captured, whether dilated or undilated was recorded.

### Glaucoma artificial intelligence tool

The Medios Glaucoma AI is a proprietary, automated deep-learning-based tool integrated on the FOP NM10 fundus camera and its development has been described elsewhere [21, 22]. In brief, the AI system includes two deep neural networks (1) a cup and disc segmentation model, an assistive network that detects the optic disc, crops the region of interest around it, outlines the disc and cup and finally outputs the vertical cup-to-disc ratio (vCDR) and (2) a binary classification model. The final output includes vCDR along with the classification of “glaucoma”, “disc suspect or high vCDR” or “no glaucoma”.

**Fig 1:**
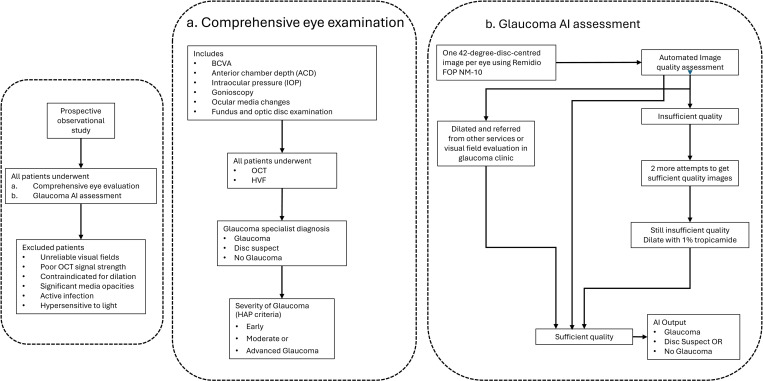
Flowchart of the Study Methodology.

The flowchart of the study methodology is presented in Fig 1.

### Sample size

Assuming an 80% sensitivity, 10% precision, 40% glaucoma prevalence, 95% confidence level and anticipating 10% of poor image quality, the estimated sample size calculated was 200 patients.

### Statistical analysis

All the data was entered into Microsoft Excel. The AI and glaucoma specialist diagnosis was categorized as “glaucoma”, “disc suspect” and “no glaucoma”. We defined referral warranted glaucoma by combining those diagnosed as “disc suspect” and “definite glaucoma”. A patient-level analysis including the diagnosis of the worse eye for the presence of referral-warranted glaucoma was used to compare the AI against the specialist diagnosis. Confusion matrix was used to compute the sensitivity and specificity of the AI system against the clinician diagnosis. Additional metrics included the positive predictive value (PPV) and the negative predictive value (NPV), and accuracy along with Wilson’s 95% Confidence Intervals (CI).

### Inclusivity in global research

Additional information regarding the ethical, cultural, and scientific considerations specific to inclusivity in global research is included in the Supporting Information (S3 Checklist in S2 File).

## Results

**Fig 2:**
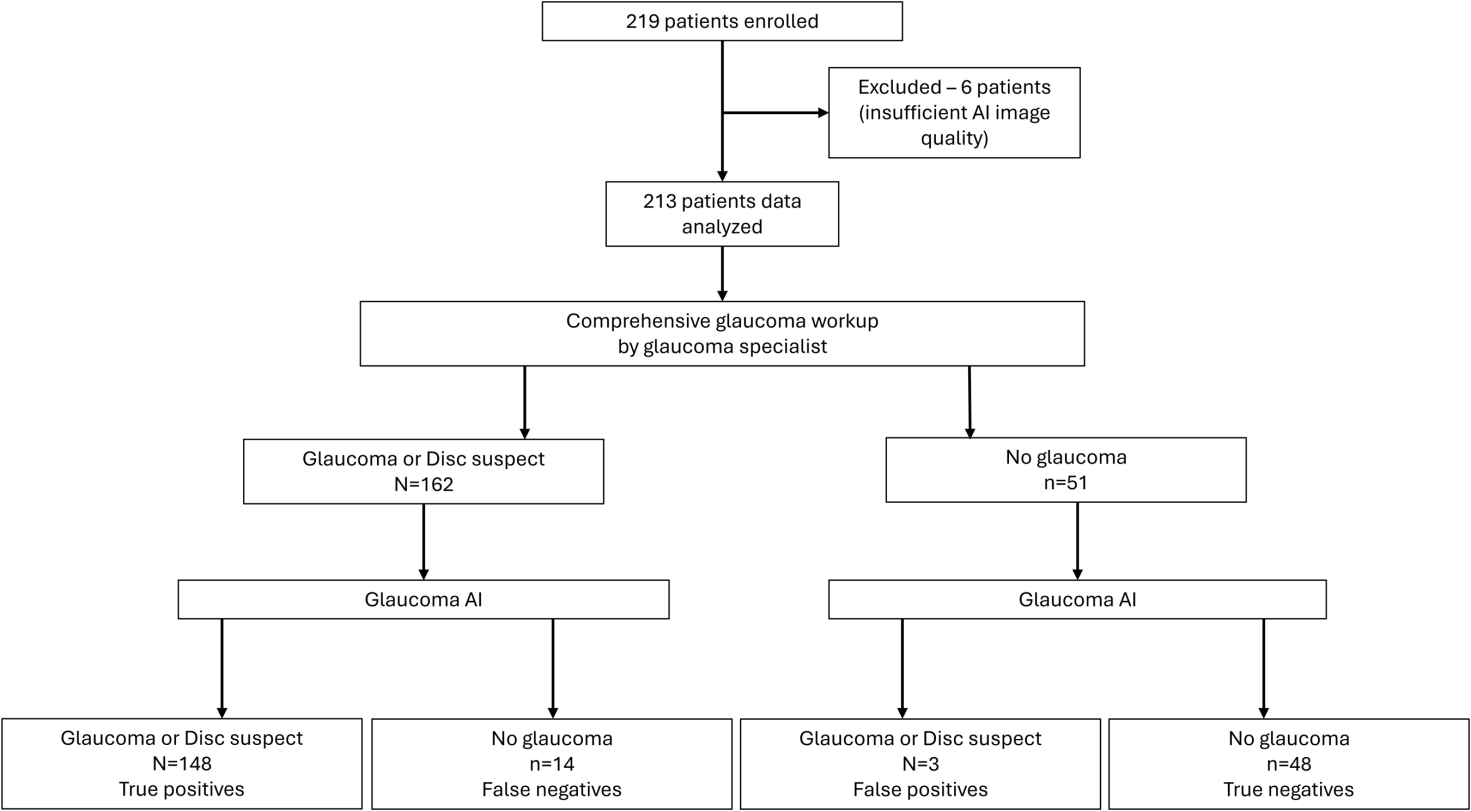
Standard for Reporting Diagnostic Accuracy (STARD) flow diagram of participant disposition: Glaucoma Artificial Intelligence against specialist diagnosis.

The study included a total of 219 patients and 6 were excluded due to insufficient AI system image quality in both eyes. We analyzed 213 participants (418 eyes) with a mean age of 55 ± 14.7 years (18–88 years). Of these, 62% (n = 131) were male, and 25% (n = 54) had a history of diabetes mellitus. Undilated images were captured in 51% (n = 108) of the patients, and the majority of the dilated images (n = 101) were captured following visual field testing when the pupil was in a predilated state. A total of 129 definite glaucoma (23 early, 31 moderate and 75 advanced glaucoma) and 33-disc suspects as diagnosed by the specialist were included. Fig 2 presents the Standard for Reporting Diagnostic Accuracy (STARD) flow diagram of participant disposition.

### Diagnostic performance of glaucoma AI against specialist diagnosis

**Fig 3:**
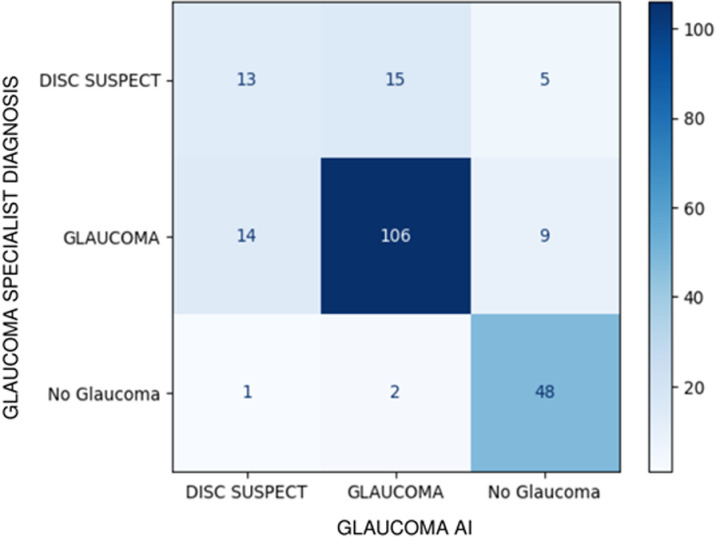
3x3 Confusion matrix: Performance of Glaucoma AI against Specialist Diagnosis based on glaucoma workup.

**Fig 4:**
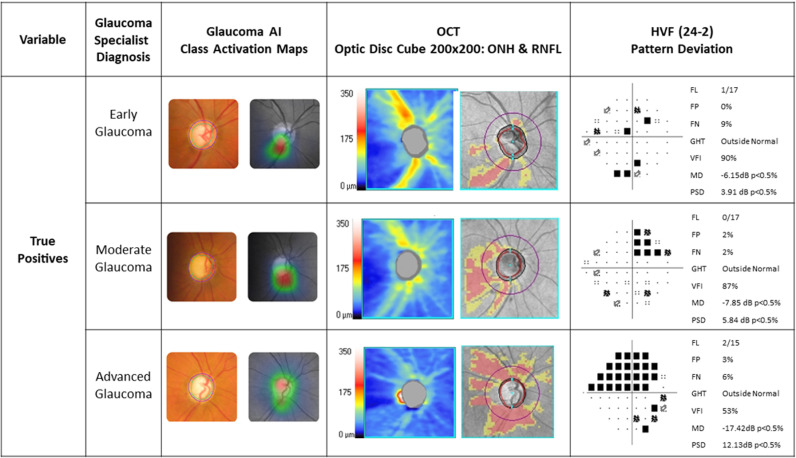
Combined Glaucoma AI and Clinical Structure-Function report based on glaucoma severities – True Positives.

**Fig 5:**
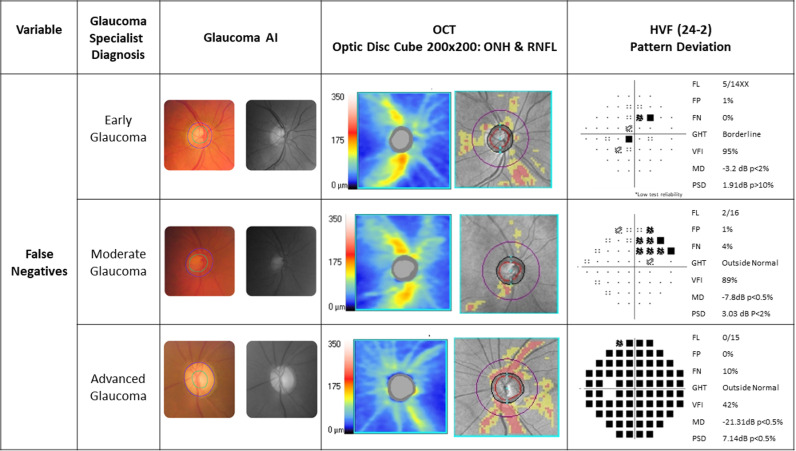
Combined Glaucoma AI and Clinical Structure-Function report based on glaucoma severities – False Negatives.

**Fig 6:**
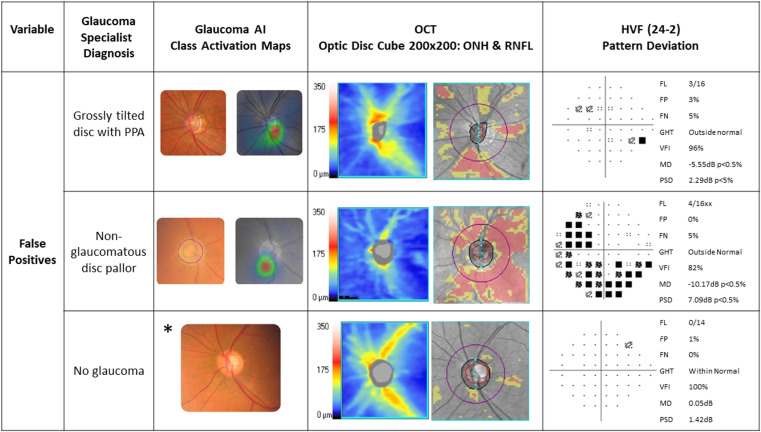
Combined Glaucoma AI and Clinical Structure-Function report based on glaucoma severities -False Positives.

The glaucoma specialist diagnosed 129 as ‘definite glaucoma’, 33 as ‘disc suspect’ and 51 as ‘no glaucoma’ based on a comprehensive glaucoma workup (Fig 3, 3x3 confusion matrix). The sensitivity and specificity of the glaucoma AI system in detecting referral warranted glaucoma (definite glaucoma + disc suspect) against the glaucoma specialist diagnosis was 91.36% (95% CI 85.93% to 95.19%) and 94.12% (95% CI 83.76% to 98.77%) respectively (Table 1, overall diagnostic performance of the glaucoma AI). Among the false negatives (n = 14), 9 were diagnosed as definite glaucoma and 5 as disc suspects by the glaucoma specialist. Among the 9 with definite glaucoma missed by AI, 3 were early, 3 moderate and 3 advanced glaucoma and the specialist diagnosed them as POAG (n = 3), PACG (n = 4) or Secondary glaucoma (n = 2). While 6 cases out the 9 had typical structural changes that were missed by the AI, 3 among the 6 had poor image quality. Coupled with poor image quality due to presence of cataract may have led to a misdiagnosis in 5 out of 9 cases, including challenging findings such as disc pallor with diffuse RNFL loss OR sloping rims. There were 3 false positives by AI. The specialist diagnoses were one each as no glaucoma, grossly tilted disc with PPA and ocular hypertension, and non-glaucomatous disc pallor. Figs 4–6 present a combined glaucoma AI with Class Activation Maps (CAMs) and clinical structure-function report (HVF and OCT) for true positives, false negatives and false positives, respectively, based on glaucoma severity levels. We looked at the output in two subsets, the elderly (>65 years) and in JOAG. In adults over 65 years old (n = 60), 53 had referral warranted glaucoma and 7 had no glaucoma. The glaucoma AI demonstrated a sensitivity of 90.57% (95% CI: 79.34% to 96.87%) in detecting referral-warranted glaucoma and all the 7 no glaucoma cases were correctly detected to have no changes. The study included 7 Juvenile Open-angle glaucoma (JOAG) and all were correctly identified as definite glaucoma by the AI system.

**Table 1:** Diagnostic accuracy of glaucoma AI in detecting referral warranted glaucoma (n = 213) against glaucoma specialist diagnosis based on comprehensive glaucoma workup.

Statistic	Value	95% CI
**Sensitivity**	91.36%	85.93% to 95.19%
**Specificity**	94.12%	83.76% to 98.77%
**Positive Predictive Value**	98.01%	94.30% to 99.59%
**Negative Predictive Value**	77.42%	65.03% to 87.07%
**Accuracy**	92.02%	87.53% to 95.28%

Referral warranted glaucoma defined as combining “definite glaucoma” and “disc suspect” by the glaucoma specialist

### Diagnostic performance of the glaucoma AI based on glaucoma severity

**Fig 7:**
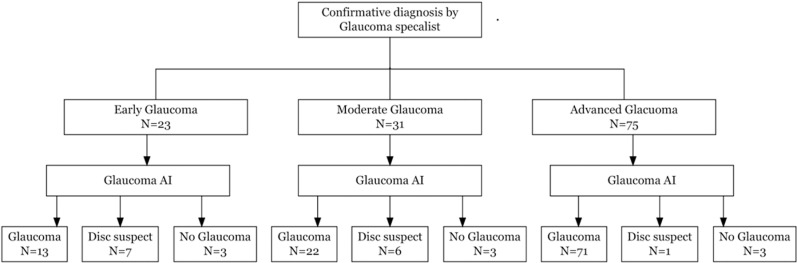
Glaucoma AI Diagnostic Performance Across Different Glaucoma Severities.

The AI performance against glaucoma specialist diagnosis for advanced glaucoma (n = 75) was higher compared to moderate (n = 31) and early glaucoma (n = 23). The diagnostic performance of the AI against glaucoma specialist diagnosis and various severities is summarized in Fig 7 & Table 2.

**Table 2:** Diagnostic accuracy of glaucoma AI against specialist diagnosis based on glaucoma severity.

Glaucoma severity	Sample	Sensitivity
**Early**	23	86.96% (66.41% to 97.22%)
**Moderate**	31	90.32% (74.25% to 97.96%)
**Advanced**	75	96.00% (88.75% to 99.17%)

### Diabetes and glaucoma

Among the patients with a history of diabetes (54 out of 213), glaucoma specialist diagnosed 83% (45 patients) to have definite glaucoma (n = 38 patients) or disc suspects (n = 7 patients). The AI could correctly identify 42 out of 45 patients with definite glaucoma or disc suspect.

## Discussion

The way to combat the burden of an asymptomatic blinding disease like glaucoma is early detection and appropriate care. Lack of awareness, complexity in diagnosis, fewer trained specialists make the initiative to tackle glaucoma blindness challenging. We need tools that can help with rapid screening of at-risk population and referral so that the available resources can be better utilized and appropriate care provided.

With increased life expectancy and ageing population, glaucoma patients will have the disease for a longer time, potentially raising the lifetime risk of glaucoma-related blindness. Peters et al evaluated the lifetime glaucoma risk in the deceased and reported approximately 1 in 6 glaucoma patients with bilateral blindness (median duration of 2 years) during their last visit and 40% experiencing unilateral blindness [26].

In the current study, we evaluated a promising and affordable solution for glaucoma screening in a real-world setting using a glaucoma AI algorithm integrated into a smartphone-based fundus camera. The performance of the AI tool to detect referral-warranted glaucoma (including definite glaucoma or disc suspects) using retinal images, showed promising results with sensitivity and specificity of 91.36% (95% CI 85.93% to 95.19%) and 94.12% (95% CI 83.76% to 98.77%) respectively, when compared to a glaucoma specialist diagnosis.

Though several AI algorithms have been developed for glaucoma screening using fundus images, most underwent validation only on retrospective datasets, leaving a glaring gap in real-world evidence [11–20]. Previous studies using this device have shown performance of this AI model for glaucoma at a refer or no-refer level [21–23]. The current study went beyond merely evaluating AI’s overall performance, delving into the AI performance in mild, moderate and advanced glaucoma, adding a layer of depth to the investigation. In this study, the AI system missed nine definite glaucoma cases, including early, moderate, and advanced stages. In advanced cases—two PACG and one steroid-induced glaucoma—were missed, these eyes wither had pallor of the disc or had poor quality disc images. These are possible limitations of AI’s ability to detect atypical changes and high reliability on the image clarity. Subtle findings in early and moderate cases, such as early rim thinning and vertical asymmetry, may have been underrecognized as well. To mitigate the FNs even further, the next version of the algorithm could be trained specifically on these challenging cases. Further evaluation in a community screening setting will also provide insights into whether there is a need to modify the threshold of the image quality algorithm to a more stringent one.

General public screening or non-targeted screening for glaucoma is not recommended, as it is not cost-effective and there is no simple screening tool or strategy. At-risk population screening has shown promise, particularly in developing countries [27,28]. In our previous validation within a vision center model, the AI’s performance was comparable to teleophthalmologist grading (general ophthalmologist and not a trained glaucoma specialist) with a sensitivity of 91% (95% confidence interval [CI]: 71%−99%) and specificity of 93% (95% CI: 89%−96%) in detecting referable glaucoma [23]. When patients were referred to the base hospital for comprehensive glaucoma evaluation, the AI’s diagnosis closely matched that of the glaucoma specialists (agreement of 80.3%), while teleophthalmologists had a higher rate of false referrals (55.3% agreement). Another validation comparing AI against image grading by glaucoma specialists (consensus grading) demonstrated a sensitivity of 94.64% (88.70% to 98.01%) [22]. Most of the false positives and negatives in both studies were primarily disc suspects (>50%). The current study specifically focusses on its ability to flag referable glaucoma that was compared against specialist diagnosis and AI’s performance at various glaucoma severities was also tested. The findings of our study are crucial, notably the AI’s heightened sensitivity for moderate to advanced disease. This is particularly significant in the developing world, considering an alarming 90% of undiagnosed cases in the communities, and among those diagnosed, 50% present with advanced glaucoma and 20% face imminent blindness at the time of presentation [29–31].

The study did note instances of misdiagnosis (false positives and negatives) and it is crucial to address these to improve accuracy further. Incorporating more data from OCT during training could also be a logical next step for improving accuracy further for early glaucoma. Nevertheless, given the annual revisit recommendation for individuals above 40, the chances of rapid progression of an early glaucoma case leading to imminent blindness remain relatively low. Hence, these AI algorithms serves an immediate public health need for screening at-risk population (those >40 years, high myopes, diabetics, family members of those with glaucoma). Flagging and referring those undetected moderate to advanced glaucoma for appropriate treatment and care would help decrease the blindness related burden and improve quality of life. For the intended application of AI in glaucoma screening, a patient-level approach is deemed more appropriate for making referral decisions. It is similar to the approach used by other US-FDA approved algorithms for diabetic retinopathy screening. AI uses images from both eyes to its advantage to allow for better sensitivity in patient-referral decisions. It is important to note that the AI does not differentiate between the severities of glaucoma.

**Fig 8:**
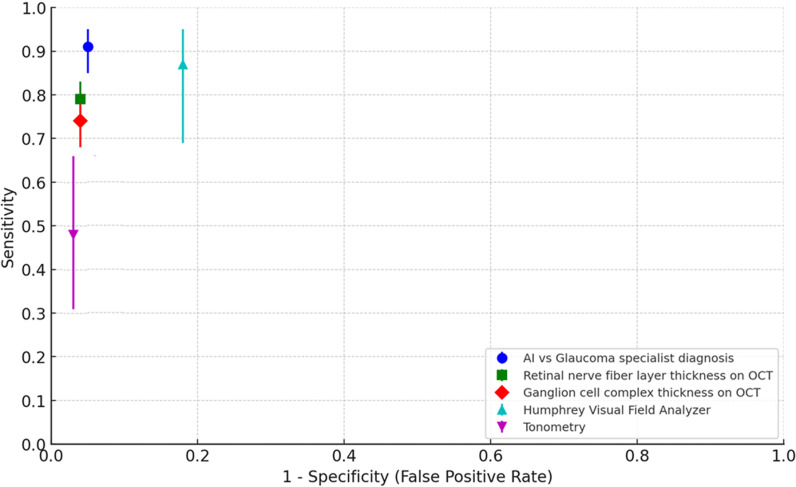
Pooled sensitivity and specificity of various studies on performance of screening tests to detect glaucoma and glaucoma AI.

The US Preventive Services Task Force (USPSTF) conducted a systematic review to assess the benefits and risks of glaucoma screening in adults [32]. The majority of the studies reviewed focused on spectral-domain OCT (29 studies) and tonometry (17 studies), with fewer studies addressing visual fields (9 studies). Fig 8 compares the pooled sensitivity and specificity from various studies analysed by USPSTF for different glaucoma screening tests with the Medios AI – Glaucoma performance based on optic disc changes. As a future scope, the AI algorithms should focus on actively incorporate such information, when available, into the system to enhance the comprehensiveness of glaucoma screening in the future.

A notable observation from our study is that 25% (54 out of 219) of the cohort had diabetes. Of these, around 83% (45 out of 54) were diagnosed with definite glaucoma or had suspicious discs as determined by the glaucoma specialist. The AI correctly detected 42 of these 45 individuals. This insight suggests that prioritizing high-risk groups, like those with diabetes, for combined screening of referable DR and glaucoma through AI-powered solutions on a singular device can be more cost-effective. There are efforts in this direction, aiming to leverage a single fundus image for multiple disease screenings, enhancing both cost efficiency and effectiveness. Interestingly, all cases of juvenile-onset open-angle glaucoma (JOAG) in our cohort (n = 7) were correctly identified by the AI tool. Given that JOAG is often underdiagnosed due to its subtle or variable presentation in younger individuals, [33] this finding is encouraging and suggests that AI may aid in earlier detection and referral in this challenging subgroup.

A key strength of this study is its prospective study design in a real-world setting, offering insights into AI’s performance across varying glaucoma severities—a dimension not explored in previous research. Additionally, standard of care formed the basis for comparison, unlike most studies which only compared AI against an image-based grading. However, limitations exist. As age advances, capturing quality images with the non-mydriatic fundus camera proves challenging due to media opacities and reduced pupil size, sometimes necessitating pupil dilation—a tangible obstacle in expansive public health screenings. While in this study, the proportion requiring dilation due to media opacity was low (4 subjects), this will require further evaluation at the population level. The limitation of this study was that nearly 50% of the subjects were dilated merely to optimize clinic workflow for follow up subjects who needed visual field monitoring and did not follow a 2-staged dilation protocol. Future scope also entails validating this model across different ethnic groups for broader global applicability.

The smartphone-based fundus camera, integrated with our glaucoma AI, distinguishes itself with affordability and ease-of-use. The device itself has been used by non-eye care professionals, and this has been established elsewhere [23]. Its capability to operate offline broadens its application scope, particularly in remote regions allowing equitable access to glaucoma care. Such an innovation stands to reshape the landscape of glaucoma screening, blending affordability with expansive accessibility.

## Conclusion

Glaucoma’s potential to cause irreversible blindness underscores the urgent need for effective screening tools. Our study presents real-world evidence supporting an AI-driven method backed by robust data, poised to address this pressing global public health challenge, particularly in resource-limited settings. The AI-based offline tool integrated on a smartphone fundus camera showed a promising performance in detecting referral-warranted glaucoma compared to a glaucoma specialist’s diagnosis. The AI showed higher accuracy in detecting advanced glaucoma followed by moderate and early glaucoma. It has the potential to enhance screening efforts, serving as a beacon of hope in combating this pervasive global issue.

## Supporting information

S1 File S1 Appendix. Exclusion criteria. **S2 Appendix.** Criteria used by the glaucoma specialists for the diagnosis and grading of glaucoma severity.
(DOCX)

S2 File Checklist. (PDF)
