# Supplementary material for: Evaluating real-world performance of an automated offline glaucoma AI on a smartphone fundus camera across glaucoma severity stages
Source: PLoS One. 2025 Jun 26;20(6):e0324883. doi: 10.1371/journal.pone.0324883 (PMC12200649; doi:10.1371/journal.pone.0324883)
Supplement: S1 File — Exclusion criteria. S2 Appendix. Criteria used by the glaucoma specialists for the diagnosis and grading of glaucoma severity. (DOCX) [file pone.0324883.s001.docx]

**S1 Supplementary Appendix 1:** Exclusion criteria

We excluded subjects with unreliable visual fields (defined as >20% rate of fixation losses and >15% false-positive and false-negative results), SD-OCT signal strength <6, deemed to have narrow angles on gonioscopy and cannot be dilated without a laser iridotomy (PI), unwilling to sign the informed consent, with acute or sudden vision loss, other causes of optic neuropathy, co-existing retinal/disc pathologies that does not allow reliable assessment of disc like vitreous hemorrhage, congenital disc abnormalities, advanced retinopathy of prematurity etc, significant media opacity precluding adequate view of the disc like advanced cataract as described below, corneal scars, condition that, in the opinion of the investigator, would preclude participation in the study (e.g., uncontrolled intraocular pressure, active eye infection, <3months post glaucoma filtering surgery, unstable medical status including blood pressure or glycemic control etc) and hypersensitive to light, known to have seizures, consuming medication that causes photosensitivity.

**S2 Supplementary Appendix 2:** Criteria used by the glaucoma specialists for the diagnosis and grading of glaucoma severity

Clinical definitions

1. Definite glaucoma: Subject clinically showing glaucomatous change with a confirmed structural (Disc and OCT) and co-relating functional change on a reliable HVF 24-2 and/or 10-2
2. No glaucoma: Subject with no clinical, structural (Disc and OCT normal) or functional change suggestive of glaucoma
3. IOP suspect

- Ocular hypertensive – IOP recording of >24 mmHg by GAT on 2 separate readings and open angles on gonioscopy
- IOP >24 mm Hg with GAT on 2 separate occasions in an eye with primary angle closure (PAC) that had undergone laser peripheral iridotomy

1. Glaucoma suspect: Subject who is neither normal nor has certain glaucoma. Glaucoma suspect included (disc suspect)

- Disc suspect:  Eyes that had optic disc or RNFL features that are highly suspicious of glaucoma that needed regular monitoring (may include pre-perimetric glaucoma) however no visual field changes

|  | Normal  (to include PACS) | Suspect  Disc suspect  (to include pre-perimetric glaucoma) | Glaucoma |
| --- | --- | --- | --- |
| Optic disc exam | Optic disc with no glaucomatous features    AND | Any one of the following    1.VCDR ≥ 0.7 and < 0.9  2. Rim width > 0.05 DD to ≤ 0.1 DD (between 5-7 o clock or 11-1 o clock)  3. Isolated retinal nerve fibre layer defect reaching upto disc  4. Disc Haemorrhage  5. Asymmetry between the two eyes >0.2    AND | Any criterion of the following:    1.vCDR >0.7  2.NRR width ≤0.1 CDR between 11–1 or 5–7 o’clock (thinning or notch)  3. Asymmetry between the two eyes >0.2  4.Disc Haemorrhage  5.RNFLD corresponding to a narrow rim or localized notch     AND |
| SD-OCT | Normal i.e no red defects    AND | Normal or abnormal OCT    AND | RNFL <1% (red) in either quadrant or clock hour map (between 5-7 and 11-1 o clock) when segmentation is reliable.    AND |
| SAP | Normal  GHT within normal limits and PSD >5% | Reliable VF that is Normal    GHT within normal limits and PSD >5%  both on 10-2 and 24-2 | Abnormal VF i.e    Reliable VF with  1.Global loss- GHT outside normal limits, PSD <5% OR  2.Localised loss- Localised superior/inferior hemifield loss that correlates with structural damage |

**Glaucoma severity (HAP criteria)**

- Early glaucoma: VF MD upto -6 dB and typical glaucomatous optic disc change as above
- Moderate glaucoma: VF MD -6 to -12 dB and typical glaucomatous optic disc change as above
- Advanced glaucoma: VF MD >-12 dB and typical glaucomatous optic disc change as above
